# Dimensions and Anatomical Variants of the Foramen Transversarium of Typical Cervical Vertebrae

**DOI:** 10.1155/2015/391823

**Published:** 2015-09-10

**Authors:** Santosh Kaur Sangari, Paul-Michel Dossous, Thomas Heineman, Estomih Phillip Mtui

**Affiliations:** ^1^Program in Anatomy and Body Visualization, Weill Cornell Medical College, Cornell University, New York, NY 10065, USA; ^2^Department of Radiology, North Shore-LIJ Health System Hofstra/North Shore-LIJ School of Medicine, 270-05 76th Avenue, New Hyde Park, NY 11040, USA; ^3^Weill Cornell Medical College, New York, NY 10065, USA

## Abstract

The study was conducted on random sample of seventy-one dried, typical cervical vertebrae (C3–C6). The data on the age, sex, and built was not available. Using vernier calipers with 0.01 mm accuracy, the anteroposterior and transverse diameters of transverse foramina and their distance from the medial margin of the uncinate process were measured bilaterally. The mean diameter of the right/left transverse foramen varied from 2.54 mm to 7.79 mm (mean = 5.55 ± 0.87 mm) and from 2.65 mm to 7.35 mm (mean = 5.48 ± 0.77 mm), respectively. The transverse foramen was less than 3.5 mm in three vertebrae on the right and two on the left. The osteocytes observed in 21.3% of specimens and the narrow transverse foramen may place patients at risk for vertebrobasilar insufficiency or thrombus formation. The mean distance of the transverse foramen from the medial margin of uncinate process is an important landmark to avoid vertebral artery laceration and was 5.0 ± 0.87 mm (range: 3.5–7.9 mm) on the right and 5.0 ± 1.0 mm (range: 3.2–7.7 mm) on the left side. No statistically significant difference was observed between the right and left sides. The accessory transverse foramina seen in 24% of vertebrae suggest duplications or fenestrations in the vertebral artery.

## 1. Introduction

The occurrence of vertebrobasilar insufficiency caused by rotation of the head has been reported due to thickened fibroligamentous structures, osteophyte formation, thyroid cartilage compression, and congenital absence of the transverse foramen [1–4]. The transverse foramina form the passageway through which the vertebral artery ascends to enter the cranium bilaterally. The compression of the vertebral artery as a result of stenosis of the transverse foramen may also lead to clinically important consequences for patients at risk. There is scanty literature available on the diameter of the transverse foramen and its relationship to the uncovertebral joint [5–7]. Therefore this study is focused on the dimensions of the transverse foramen and the distance of the transverse foramen from the uncovertebral joint in typical cervical vertebrae (C3–C6) in American population and also elucidates the anatomical anomalies, which place patients at risk.

## 2. Materials and Methods

Seventy-one dried complete cervical vertebrae from C3–C6 were obtained from the Program in Anatomy and Body Visualization, Department of Radiology, Weill Cornell Medical College, New York. The data on age, sex, race, and built of the individuals from which these vertebrae were derived was not available. The study was conducted on a random sample of individual typical cervical vertebrae. It was not possible to specify if the vertebra is C3/C4/C5 or C6.

**Figure 1:**
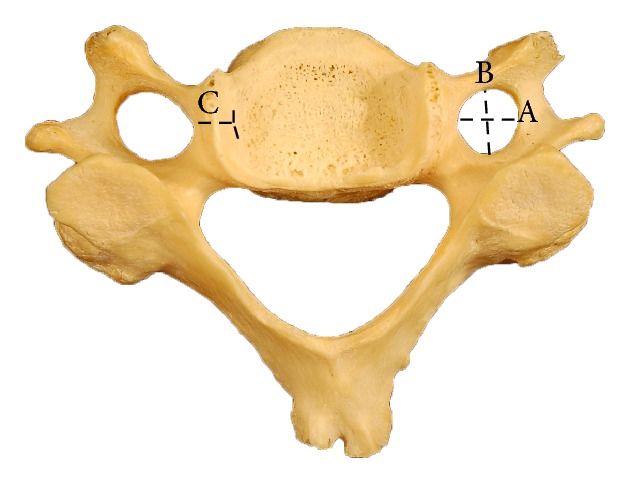
Typical cervical vertebra showing measurements of the transverse foramen: A: transverse diameter, B: anteroposterior diameter, C: distance from the medial margin of the uncinate process to the medial margin of the transverse foramen.

Using a VWR digital stainless steel vernier caliper with 0.01 mm accuracy, the anteroposterior and transverse diameters of transverse foramina were measured bilaterally. The caliper was placed within each foramen and the widest transverse and anteroposterior diameters were measured. The mean of the transverse and anteroposterior diameter of each foramen was calculated to estimate the mean diameter of each transverse foramen. The distance between the medial margin of the uncinate process and the medial end of the transverse foramen was estimated with the help of vernier calipers (Figure 1).

Statistical evaluations were performed for each measurement. The mean, standard deviation, a paired comparisons *t*-test, and *P* value were performed to determine if there was a significant difference between the right and left sides of C3–C6 vertebrae.

It was also qualitatively recorded if the specimen had osteophytes and whether or not the osteophytes were impinging on the transverse foramen. Significantly anomalous transverse foramina were documented and photographed.

## 3. Results

The foramen transversarium in the transverse processes of typical cervical vertebrae showed a wide difference in diameter in individual vertebrae. Several cervical vertebrae showed osteoporotic changes with osteophytes projecting from the body and the pedicles.

The transverse diameter of right transverse foramen showed wide variation from 2.0 mm to 8.65 mm with a mean of 5.69 ± 1.04 mm, whereas the anteroposterior diameter of right transverse foramen varied from 2.19 mm to 7.21 mm with mean diameter of 5.17 ± 0.89 mm. The left transverse foramen had a mean transverse diameter of 5.87 ± 0.89 mm (range = 2.62 mm–7.89 mm) and a mean anterior-posterior diameter of 5.13 ± 0.79 mm, ranging from 2.51 to 6.81 mm (Table 1).

Although there was remarkable difference between the transverse diameter (*t*-test of difference = 0 (versus not =): *T*-value = 0.56, *P* value = 0.57, and DF = 144) and anteroposterior diameter (*t*-test of difference = 0 (versus not =): *T* value = 0.27, *P* value = 0.79, and DF = 145) of transverse foramen on right and left side, this difference was not found to be statistically significant.

**Figure 2:**
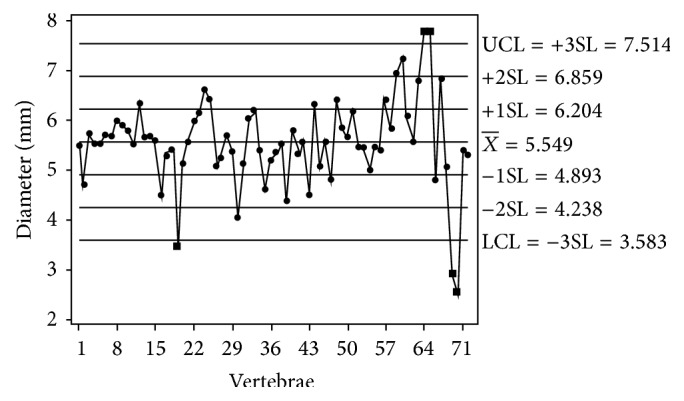
Scatter plot of the mean diameter of right sided transverse foramina.

**Figure 3:**
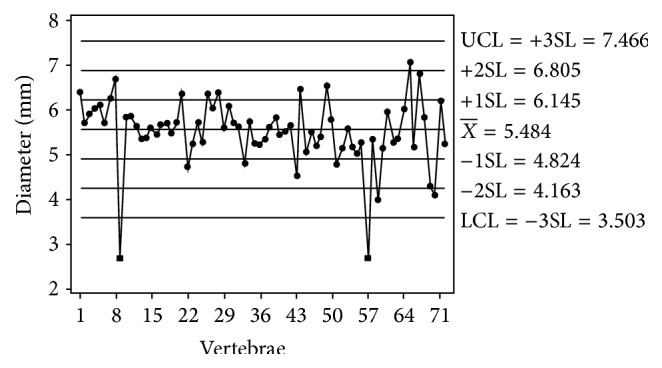
Scatter plot of the mean diameter of left sided transverse foramina.

The mean diameter of transverse foramen showed wide variation in individual typical cervical vertebrae. The right transverse foramen varied from 2.54 mm to 7.79 mm with a mean of 5.55 mm (standard deviation = 0.87) (Figure 2, Table 1) and the mean diameter of left transverse foramen varied from 2.65 mm to 7.35 mm with a mean of 5.48 mm (standard deviation = 0.77) (Figure 3, Table 1). However, there was no significant statistical difference between the mean diameter of the transverse foramen of right and left side (*t*-test of difference = 0 (versus not =): *T* value = 0.47, *P* value = 0.64, and DF = 138).

The vertebrae characterized by significant narrowing of the transverse foramen were cataloged by grouping the vertebrae for which the dimensions were smaller than one standard deviation away from the mean dimension. With this method we found that most of the transverse foramina of cervical vertebrae were within ±1 SD. There were 20 vertebrae where the diameter of the transverse foramen was ≤ 1 SD away from mean, out of which in 5 vertebrae the difference was ≤ 3 SD from mean. The mean diameter of transverse foramen was smaller than 3.5 mm in 3 vertebrae on right and in 2 vertebrae on the left side (Figures 2 and 3).

The mean distance of the transverse foramen from the medial margin of the uncinate process was 5.0 ± 0.87 mm (range: 3.5–7.9 mm) on the right side and 5.0 ± 1.0 mm (range: 3.2–7.7 mm) on the left side.

**Figure 4:**
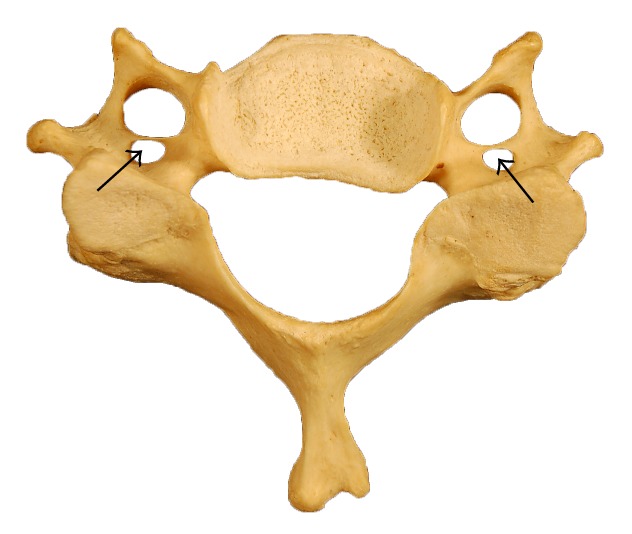
Typical cervical vertebra showing accessory transverse foramina bilaterally by arrows.

The seventeen vertebrae studied (24%) contained accessory transverse foramen with no preference to right or left side. The transverse process of one vertebra showed an accessory transverse foramen on both sides, behind the main transverse foramen (Figure 4). The accessory transverse foramina were considerably narrow and therefore were not measured (Figure 4).

**Figure 5:**
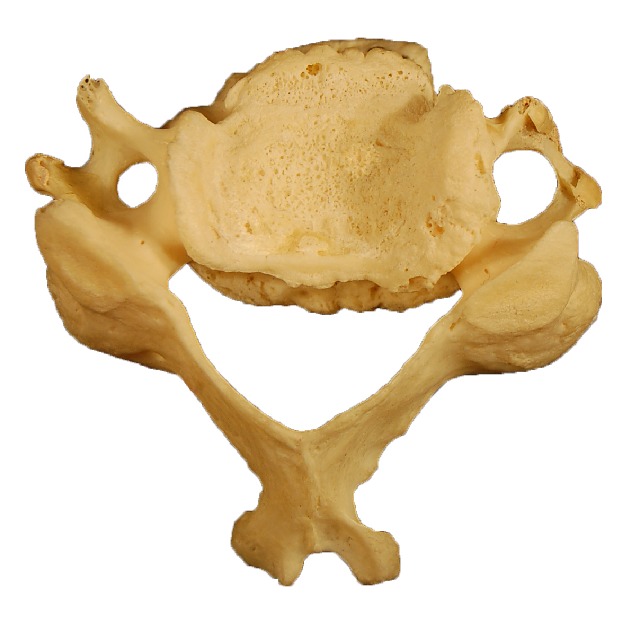
Typical cervical vertebra showing osteophytes on the body and transverse foramina of different sizes on the two sides.

While performing the measurements on the transverse foramen, it was qualitatively recorded if the specimen had an osteophyte and whether or not the osteophytes were impinging on the transverse foramen. The fifteen vertebrae out of 71 studied (21.1%) showed osteophytes with no preference on right/left side (Figure 5) and seven vertebrae (10.1%) used for study had osteophytes, which impinged on the transverse foramen.

## 4. Discussion

The two vertebral arteries are solely responsible for posterior circulation of the brain. The tortuous course of vertebral artery and rarely medial position of transverse foramen in relation to the joint of Luschka may result in life-threatening iatrogenic injury following cervical decompression [8, 9].

The two vertebral arteries are supposedly unequal in size in about 75% of cases. One of them may be extremely narrow, more so on the right side in 10% of cases [10]. In the present study, the mean diameter of the right transverse foramen varied from 2.54 mm to 7.79 mm in diameter (mean = 5.55 ± 0.87 mm), whereas the mean diameter of left transverse foramen varied from 2.65 mm to 7.35 mm (mean = 5.48 ± 0.77 mm). Although there was no statistically significant difference between the dimensions of right and left side, there remains a considerable variation in the diameter of the transverse foramen on left and right side in the same vertebra in C3–C6 vertebrae. According to the literature, the width of the transverse foramen increased from C3 to C5 vertebra (5.5  ± 0.4 mm at C3, 5.7 ± 1.0 mm at C4, 5.9 ± 0.7 mm at C5) and then decreased to 5.7 ± 0.7 mm at C6 [6].

Extreme narrowing of transverse foramen has not been reported in the literature. In the present study, the transverse foramen was less than 3.5 mm in 3 vertebrae on the right side and 2 vertebrae on the left side (Figures 2 and 3). Bow hunter's stroke is a symptomatic vertebrobasilar insufficiency caused by stenosis or occlusion of the vertebral artery with head rotation [11, 12]. It is a common finding on angiography that head rotation produces stenosis or occlusion of a contralateral vertebral artery. The narrowing of the transverse foramen may predispose patients to vertebrobasilar insufficiency and thrombus formation especially with head rotation.

The demonstration of accessory transverse foramen (Figure 4) reported in this study represents further the clinical importance of abnormal transverse foramen morphology. Duplication of extracranial vertebral artery has been reported in the literature [13–16]. Vertebral artery develops from a fusion of longitudinal anastomosis that links second to sixth cervical intersegmental arteries. Most of the intersegmental arteries regress except the seventh which forms the origin of vertebral artery. Failure of occlusion of intersegmental arteries may be responsible for duplication/fenestrations of vertebral artery. A duplicate vertebral artery may potentially serve to protect patients against ischemic attacks to the brain and provide collateral blood flow to the basilar artery. However, fenestrated vertebral arteries have been demonstrated histologically to be weak with irregular elastic fibers in the vessel wall [16]. Fenestrated/double vertebral arteries may carry more risk of thrombus formation and embolization leading to severe transient ischemic attacks.

To avoid vertebral artery injury during anterior cervical disc surgery, the medial margin of the uncovertebral joint may be the safe landmark [7]. In the present study, the mean distance of the transverse foramen from the medial margin of the uncinate process was 5.0 ± 0.87 mm (range: 3.5–7.9 mm) on the right side and 5.0 ± 1.0 mm (range: 3.2–7.7 mm) on the left side. There may be higher risk of vertebral artery laceration particularly during lateral decompression to resect osteophytes from the uncinate process. It is suggested that the lateral decompression be completed under direct visualization with opening of the anterior walls of the transverse foramen and the lateral retraction of the vertebral artery [5].

The present study showed osteophytes with no preference on right/left side in 21.3% of vertebrae studied and out of which approximately half of them were impinging on the transverse foramen. The osteophytes covering the transverse foramina may force the vertebral artery to meander around these obstructions causing narrowing through external compression and are potential sites of trauma to the vertebral artery [17].

## 5. Conclusion

Injury to the vertebral artery during anterior operative intervention in the subaxial cervical spine may give rise to the catastrophic iatrogenic complications. The study reports variations in the dimensions of the transverse foramina in a random sample of the typical cervical vertebrae (C3–C6), which allow the passage of the vertebral artery. The transverse foramen was narrow in a significant number of specimens. Additionally, the osteophytes seen in a large population of specimens which especially were seen impinging on the transverse foramen may be responsible for vertebral artery compression and trauma. Useful morphometric data is provided to assist the surgeon to prevent the vertebral artery damage. The medial margin of the uncovertebral joint is a safe landmark to avoid vertebral artery injury during anterior surgical approaches to the cervical spine. The accessory transverse foramina seen in the study suggest fenestrations or duplications in the vertebral artery.

## Figures and Tables

**Table 1:** Dimensions of transverse foramina of C3–C6 vertebrae.

Dimension	Range (mm)	Mean (mm) ± S.D.
Transverse diameter		
Right	2.00–8.65	5.69 ± 1.04
Left	2.62–7.89	5.87 ± 0.89
Anteroposterior diameter		
Right	2.19–7.21	5.17 ± 0.89
Left	2.51–6.81	5.13 ± 0.79
Mean diameter		
Right	2.54–7.79	5.55 ± 0.87
Left	2.65–7.35	5.48 ± 0.77
Distance from medial border of uncinate process to transverse foramen		
Right	3.5–7.9	5.0 ± 0.87
Left	3.2–7.7	5.0 ± 1.0
